# Critical evaluation of linear regression models for cell-subtype specific methylation signal from mixed blood cell DNA

**DOI:** 10.1371/journal.pone.0208915

**Published:** 2018-12-20

**Authors:** Daniel W. Kennedy, Nicole M. White, Miles C. Benton, Andrew Fox, Rodney J. Scott, Lyn R. Griffiths, Kerrie Mengersen, Rodney A. Lea

**Affiliations:** 1 ARC Centre of Excellence for the Mathematical and Statistical Frontiers, Queensland University of Technology, Brisbane, Australia; 2 School of Mathematical Sciences, Queensland University of Technology, Brisbane, Australia; 3 Genomics Research Centre, Institute of Health and Biomedical Innovation, Queensland University of Technology, Brisbane, Australia; 4 Florey Department of Neuroscience and Mental Health, Melbourne, Australia; 5 Centre for Information-Based Medicine, Hunter Medical Research Institute, Newcastle, Australia; Institut de Genomique, FRANCE

## Abstract

Epigenome-wide association studies seek to identify DNA methylation sites associated with clinical outcomes. Difference in observed methylation between specific cell-subtypes is often of interest; however, available samples often comprise a mixture of cells. To date, cell-subtype estimates have been obtained from mixed-cell DNA data using linear regression models, but the accuracy of such estimates has not been critically assessed. We evaluated linear regression performance for cell-subtype specific methylation estimation using a 450K methylation array dataset of both mixed-cell and cell-subtype sorted samples from six healthy males. CpGs associated with each cell-subtype were first identified using *t*-tests between groups of cell-subtype sorted samples. Subsequent reduced panels of reliably accurate CpGs were identified from mixed-cell samples using an accuracy heuristic (*D*). Performance was assessed by comparing cell-subtype specific estimates from mixed-cells with corresponding cell-sorted mean using the mean absolute error (MAE) and the Coefficient of Determination (*R*^*2*^). At the cell-subtype level, methylation levels at 3272 CpGs could be estimated to within a MAE of 5% of the expected value. The cell-subtypes with the highest accuracy were CD56^+^ NK (*R*^*2*^ = 0.56) and CD8^+^T (*R*^*2*^ = 0.48), where 23% of sites were accurately estimated. Hierarchical clustering and pathways enrichment analysis confirmed the biological relevance of the panels. Our results suggest that linear regression for cell-subtype specific methylation estimation is accurate only for some cell-subtypes at a small fraction of cell-associated sites but may be applicable to EWASs of disease traits with a blood-based pathology. Although sample size was a limitation in this study, we suggest that alternative statistical methods will provide the greatest performance improvements.

## Introduction

DNA methylation is characterized by the modification of DNA by the chemical addition of a methyl group (CH_3_). To date, the most investigated methylation mechanism occurs at Cytosine followed by a Guanine base, called a CpG site. There is great interest in the effects of environmental exposures including diet [1], smoking [2] and stress [3] on DNA methylation. Associations between methylation and disease risk, for example, metabolic syndrome [4] and type-2 diabetes [5] have also been examined. Studies on the interaction of age and methylation [6] have demonstrated that the effect of age on methylation level differs between cell-subtypes and between tissue-type [7,8]. Research has also addressed the role of methylation patterns in mammalian development [9], and cell-differentiation [10,11], and it has been shown that the cell lineage of blood cells can be inferred from methylation patterns measured in cell-sorted samples [12]. Cell-sorted samples were found to cluster together based on cell-subtype rather than subject, indicating cell-subtype methylation is stable between subjects.

Whole blood consists of a number of different types of nucleated leukocytes that proportionately contribute to the overall methylation signal observed. Variation in methylation among constituent cell-subtypes has motivated the development of methods to estimate methylation levels from heterogeneous tissues such as whole blood. Laboratory-based approaches for isolating components, such as flow-sorting in blood and laser-capture microdissection [13] for solid tissue, tend to be financially prohibitive. Additionally, there may not be *a priori* knowledge of which cell-subtypes are associated with a phenotype of interest, so separation of the tissue into all constituent cell-subtypes with a possible association is not plausible.

The estimation of cell-subtype methylation signals from heterogeneous samples with the aid of cell-subtype composition information, called here Cell-subtype Specific Methylation Estimation (CSME), has received little attention to date in the literature. This task is distinguished from the traditional Epigenome-Wide Association Study (EWAS), whereby the effect or association of a phenotype or disease on methylation is inferred, correcting for possible cell-subtype related variation (see the recent review by Titus et al. [14] for examples). Rather, with CSME the focus is on estimating the cell-type specific methylation level without any explicit relation to a phenotype or disease. Another important distinction is the difference between CSME and the proportion estimation algorithms such as the constrained projection method [15]. The goal of proportion estimation is to use observed whole blood methylation levels from cell-type associated CpGs to estimate the relative proportions of the component cell-subtypes in samples, however the goal of CSME is to use estimates of relative cell-type proportion to estimate the cell-type level methylation.

A linear regression approach has been developed for discriminating between two cellular components (neuronal and glial cells) in the methylation signal from brain tissue, and it has been suggested that this method could be extended to more than two cell-subtypes via the aggregation of non-target cell-subtypes [16]. Population-Specific Expression Analysis (PSEA) [17] is another linear regression approach for brain tissue but this was designed for gene expression, not methylation data. Since both these methods were applied to brain tissue and one was for gene expression, their performance on blood methylation, in terms of accurate estimation of cell-type methylation, is not known.

This paper aims to determine the utility of linear regression for CSME. Using empirical methylation data this paper critically analyses the performance of linear regression to estimate cell-subtype methylation patterns from mixed (whole) blood cell samples. The evaluation specifies the CpGs as well as cell-subtypes and groupings where linear regression yields reasonable estimation performance, within a specified level of error.

## Methods

The focus of this paper was to quantify the accuracy of the linear regression approach for CSME by comparing Linear Regression (LR) estimates from mixed-cell samples to cell-sorted estimates derived from purified, cell-sorted samples collected from the same subjects.

Analyses were performed using publicly available 450K methylation data derived from 6 healthy males [13] (GEO Accession Number: GSE35069) of age range 38±13.6 years. The Methylation data was comprised of mixed-cell (whole) blood as well as 7 different blood cell types, namely; Eosinophils (Eos), Neutrophils (Neu), CD4^+^ and CD8^+^T cells, CD19^+^B cells, CD56^+^ Natural Killer (NK) cells, and CD14^+^ monocytes (Mono). Our calculations also included flow-sorted estimates of cell-subtype proportions, which were found in the Supplementary Information of [13] (https://doi.org/10.1371/journal.pone.0041361.s004).

CSME performance was also analysed for groups of blood cell-subtypes based on major lineages (lymphocytes and myeloids), called lineage groupings. For the dataset used in this paper, it has been shown that the cell-sorted methylation data clustered consistently into major hematopoietic cell lineages; lymphocytes (called Lymphocyte-I here), and myeloids (called Myeloid-I here) [13]. It was also noted that CD19^+^B seemed to be distinguished from other cell-subtypes in Lymphocyte-I, so a second subset not containing CD19^+^B called Lymphocyte-II was studied, as well as the PanT grouping and the Granulocyte (called here Myeloid-II) grouping.

Performance was first analysed at the epigenome-wide level. Subsequently, a set of criteria were used to identify a robust CpG panel for each cell-subtype and lineage grouping, and then the performance of linear regression was analysed over these panels.

### Defining cell-subtype methylation signal by the simple linear regression slope

In Linear Regression CSME, estimating cell-subtype methylation signal centres on the simple linear relationship (slope) of the individual level cell-subtype proportion and the mixed-cell methylation level at a given CpG. The procedure takes as inputs a) methylation profiles sampled from heterogeneous mixtures of cell-subtypes and b) estimates of the proportions of the constituent cell-subtypes. In this study the latter were measured by flow cytometry but these values could also be inferred from cell-specific reference CpGs [15]. The outputs are inferences about the underlying cell-subtype methylation profiles.

The degree of methylation for a given sample at a given CpG *j* can range between 0 (unmethylated) and 1 (methylated), and is referred to as a beta-value. These values can be viewed as a measure of the probability that a strand of DNA in the sample will be methylated at that CpG.

The mixed-cell beta-value is assumed to be a linear combination of the constituent cell-subtype beta-values, weighted by their respective proportions of the cell-subtypes. Therefore, the expected observed methylation level for given sample and CpG can be represented as the linear combination of the set of the static cell-subtype methylation values weighted by their respective cell-subtype proportions. This is consistent with another modelling approach [16] taken when only two cell-subtypes were present. Linear Regression CSME for a target cell-subtype against the other constituents requires agglomeration of the other cell-subtypes into a single group by adding their proportions together.

Let *x*_*jk*_ be the underlying methylation level of the target cell-subtype at site *j* and *x*_*j*(−*k*)_ is the methylation of all other cell-subtypes combined. Therefore the beta-value for sample *i* and CpG *j*, *y*_*ij*_ can be expressed as:

$$y_{ij}=p_{ik}x_{jk}+(1-p_{ik})x_{j(-k)}+e_{ij},\;e_{ij}∼Normal(0,\sigma_{j}).$$

Taking *β*_0*jk*_ = *x*_*j*(−*k*)_ and *β*_1*jk*_ = *x*_*jk*_ − *x*_*j*(−*k*)_ one can formulate the above equation as a simple linear regression model:

$$y_{ij}=\beta_{0jk}+\beta_{1jk}p_{ik}+e_{ij}.$$

The full derivation of the above equation is given in S1 File, where the main assumptions are that the whole blood methylation *y*_*ij*_ is equal to the average of the cell-type methylation levels, weighted by the cell-type proportions, and that the unmodelled error is normally distributed. As shown by the above equation, the difference in methylation between the target cell-subtype and other cell-subtypes in the sample *β*_1*jk*_ can be estimated by regressing the mixed-cell methylation data on the target cell-subtype proportion. The linear regression model is easily extended to target larger groupings of cell-subtypes by adding their proportions together.

While beta-values are bounded by 0 and 1, the choice of a Normal noise distribution for beta-value methylation is consistent with several previous methods [15,16]. It is common practice to use logit-transformed M-values which are unbounded and thus tend to have more Normal noise distributions, however the logit-transform is non-linear and therefore could not be viewed as the weighted average of underlying cell-type methylation levels.

### Deriving expected CpG values from cell-sorted data

In order to measure the performance of the method, ground-truth reference (or expected) values needed to be calculated for each CpG, called cell-sorted estimates. These were calculated using cell-sorted data for the 6 subjects and for the 7 different blood cell-subtypes [13]. Corresponding cell proportion estimates measured independently with Fluorescence-Activated Cell Sorting (FACS) were used in the calculations. The expected value for the difference in methylation of a cell-subtype *k* from the other constituent cell-subtypes was calculated as the difference in means:

$${\bar{x}}_{jk}-{\bar{x}}_{j(-k)}=\frac{1}{N}\sum_{i=1}^{N}x_{ijk}^{*}-\frac{1}{N(K-1)}\sum_{i=1}^{N}\sum_{q\neq k}x_{ijq}^{*}$$

where $x_{ijk}^{*}$ is the beta-value of cell-subtype *k* in sample *i* at CpG *j*, and ${\bar{x}}_{jk}$ and ${\bar{x}}_{j(-k)}$ are the cell-sorted means at CpG *j* for *k* and the remaining cell-subtypes respectively. For cell-subtype groupings such as Lymphocyte-I and Myeloid-I, the cell-sorted estimate was calculated similarly.

$${\bar{x}}_{jC}-{\bar{x}}_{j(-C)}=\frac{1}{Nn_{C}}\sum_{i=1}^{N}\sum_{q\in C}x_{ijq}^{*}-\frac{1}{N(K-n_{c})}\sum_{i=1}^{N}\sum_{q\notin C}x_{ijq}^{*}$$

where *C* is the set of cell-subtypes contained within the grouping, and *n*_*C*_ is the number of cell-subtypes within the grouping.

### Selecting robust CpG panels for estimation

The use of the proposed linear regression model centres around inference about cell-specific methylation at CpGs that are *a priori* assumed to be differentially methylated among cell-subtypes. We identified these cell-associated CpG panels for each cell-subtype using a two-sided, two sample pooled *t*-test of difference of means. The cell-sorted data for base cell-subtypes was divided into two groups; the samples purified to the target cell-subtype (via Fluorescence-Activated Cell Sorting), and the samples not purified to the target cell-subtype. For lineage groupings, the two groups in the *t*-test were discriminated as being within the lineage grouping or not within the lineage grouping. The Benjamini-Hochberg method was applied to control the false discovery rate (FDR) [18]. The result was a cell-sorted CpG panel with the smallest p-values and a nominal FDR of 10^−4^.

A Cell-subtype associated CpG was deemed accurately estimated if the cell-subtype methylation estimate was sufficiently close to the cell-sorted estimate, and we developed an accuracy heuristic (*D*) which would be used to discard CpGs with a significant discrepancy, resulting in a reduced, robust CpG panel for each cell-subtype, comprised of CpGs which are both cell-associated, and can be accurately estimated.

The accuracy heuristic was calculated with a calibrated estimate. It was assumed that LR estimates should be accurate, although it was permissible for there to be some level of position shift and inflation, since cell-type methylation profiles could be correlated, and that a post-hoc statistical calibration phase could easily be implemented to correct the estimates (see S2 File for a simulated demonstration of this). For statistical calibration, we used a linear regression between the LR estimates and the cell-sorted estimates. The LR estimate was transformed into a calibrated estimate which had a slope of 1 and an intercept of 0 when regressed against the cell-sorted estimate. The transformation used was:

$$\mathrm{Calibrated}\;\mathrm{cell}‑\mathrm{subtype}\;\mathrm{methylation}\;\mathrm{estimate}=\frac{\mathrm{cell}‑\mathrm{subtype}\;\mathrm{methylation}\;\mathrm{estimate}-\hat{\alpha }}{\hat{\beta }}$$

where $\hat{\alpha }$ and $\hat{\beta }$ are the intercept and slope estimates when the LR estimates are regressed against the cell-sorted estimates.

The calibrated estimate was compared with the cell-sorted estimate to give an error value for each of the CpGs in the candidate CpG panel. The accuracy heuristic was calculated by dividing by the standard error of the calibrated estimate:

$$D=\frac{\mathrm{Calibrated}\;\mathrm{cell}‑\mathrm{subtype}\;\mathrm{methylation}\;\mathrm{estimate}-\mathrm{Cell}‑\mathrm{sorted}\;\mathrm{Estimate}}{SE(\mathrm{Calibrated}\;\mathrm{cell}‑\mathrm{subtype}\;\mathrm{methylation}\;\mathrm{estimate})}$$

where

$$\mathrm{SE}(\mathrm{Calibrated}\;\mathrm{cell}‑\mathrm{subtype}\;\mathrm{methylation}\;\mathrm{estimate})=\frac{1}{\left|\hat{\beta }\right|}\mathrm{SE}(\mathrm{cell}‑\mathrm{subtype}\;\mathrm{methylation}\;\mathrm{estimate}).$$

The accuracy heuristic *D* is a standardized estimate of the discrepancy between the calibrated estimate and the cell-sorted estimate.

It was reasonable to consider that the standard Student’s-*t* distribution with (*N*−2) degrees of freedom would be the distribution of *D* when there was no difference between the two estimates (see S3 File for more detail). This is because *D* is equal to a linear regression slope up to a sign. Therefore, CpGs where

Pr(|T|≥|D|)<0.05

for a *t*(*N*−2) distributed random variable *T* were rejected as too inaccurately estimated. The result was a reduced, robust panel of accurately estimated CpGs from the candidate panels.

### Assessing estimation performance of cell-specific CpGs

In this analysis, the estimation performance was assessed by calculating the deviation of the cell-subtype methylation estimate from the cell-sorted estimate. Specifically, the mean absolute error (MAE) between the calibrated cell-subtype methylation estimate and the cell-sorted estimates was calculated for all CpGs in the robust CpG panels. The mixed-cell error (MCE), equal to the absolute value of the cell-sorted estimate, was used as a measure of the base level of error from which cell-subtype methylation estimation could improve. This is because it is the error of estimating the difference in mean methylation between a given cell-type and the others as 0. For an entire panel, the MCE values for the entire panel were averaged to give a panel wide estimate of baseline error, called the Mean Mixed-cell Error (MMCE).

When evaluating estimation accuracy of CpGs it was also considered that cell-subtype methylation estimates only have to correlate with their corresponding cell-sorted estimates, since a post hoc centering and calibration phase can be adopted as above to obtain a properly centred and scaled estimate. The Coefficient of Determination *R*^*2*^ in simple linear regression was used as the measure of accuracy. *R*^*2*^ can have values ranging from 0 to 1, with 0 indicating no linear correlation and 1 indicating perfect linear correlation between LR estimates and cell-sorted estimates.

All analysis completed using the statistical programming language R[19]

### Validation of robust CpG panels with supplementary dataset

A supplementary dataset (GSE Accession number: GSE88824) was used to validate the robust CpG panels found using the original dataset. This dataset contained mixed-cell data as well as cell-sorted sample data for all cell-subtypes except for Eosinophils, so the Eosinophil as well as Myeloid-II panels were not validated. Subjects sampled consisted of 5 females and 3 males, and mean age 39. Robust CpG panels were found for the validation dataset and the number and percentage of overlap between the original panel and the validation panels was calculated. The expectation was that if a large proportion of CpGs in the original were also found in the corresponding validation panel, then this would indicate that the method and therefore panel are robust over different datasets.

## Results

### Linear regression leads to poor estimation performance for estimated cell-specific methylation at the epigenome-wide level

For all cell-subtypes and lineage groups, linear regression was used with mixed blood cell data to estimate cell-subtype specific methylation, characterised by the difference between methylation of the target cell-subtype and the other constituents. Linear regression produced an estimate of this difference at each locus, called the Linear Regression (LR) estimate. Independently, the difference was also calculated using the cell-sorted data, delivering a ground-truth, cell-sorted estimate, to which the LR estimate is compared.

**Fig 1:**
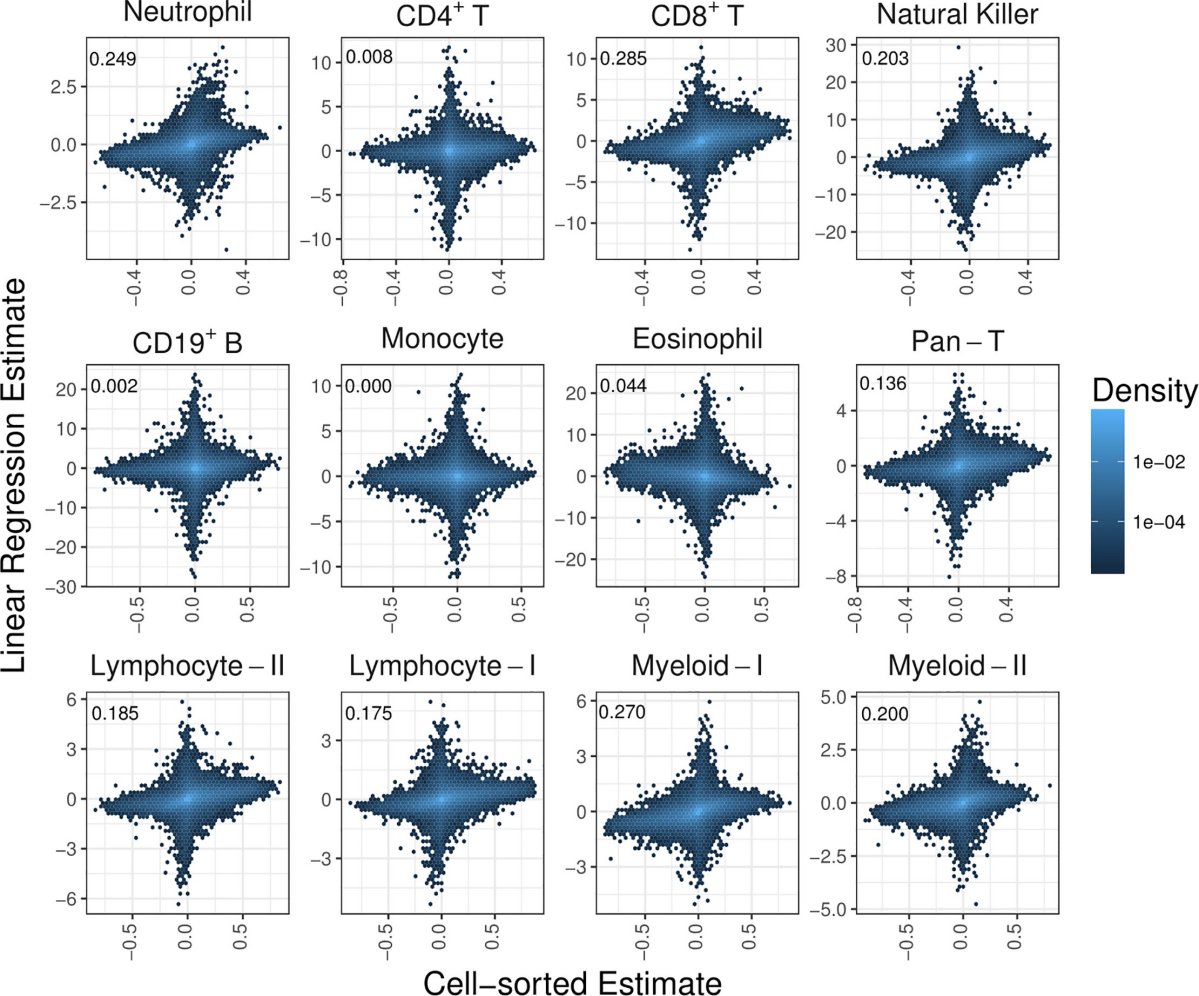
Comparison of LR estimate versus cell-sorted estimates. Hexagon plots of LR estimate of cell-type methylation difference on the vertical axis obtained from mixed-cell data is compared with the cell-sorted estimate of cell-type methylation difference on the horizontal axis obtained independently from cell-sorted data for all measured CpGs. The shading of the hexagonal bin indicates the density of CpGs in that bin. This comparison is made for each cell-subtype and lineage grouping.

LR estimates (observed) and cell-sorted estimates (expected) values were compared for 445,603 CpGs. Results showed a bi-directional pattern at the epigenome-wide level (Fig 1). Overall, this pattern was indicative of many CpGs having either small cell-sorted estimates corresponding with large cell-subtype methylation estimates (vertical axes), or small cell-subtype methylation estimates and large cell-sorted estimates (horizontal axes). The pattern was indicative of poor estimation performance for the majority of CpGs across the epigenome. Although CS estimates and methylation differences must lie between -1 and +1, since the LR estimates are regression coefficients there was no constraint for them to fall within this range, and so there were many instances where this was the case.

### Robust CpG panels cover at most 23% of the observed epigenome

For the single cell-subtypes the total number of cell-subtype associated CpGs deemed accurately estimated was 33916, corresponding to 7.6% of the observed epigenome (Table 1). A small number of CpGs were discarded based on a high *D-*heuristic (0.0–5.7% of candidate CpGs). The number of accurately estimated CpGs varied substantially by cell-subtype, with CD8^+^T having the smallest number (1871) and CD19^+^B having the largest (12973). The largest robust CpG panels were for the primary lineage groupings Lymphocyte-I and Myeloid-I, whereby 103,112 CpGs (23% of the observed epigenome) could distinguish these groups.

**Table 1:** Estimation performance over the robust panels for cell-subtype and lineage groupings.

Panel	Panel Size	MAE	MMCE	Observed versus Expected (*R*^*2*^)	Number (%) with AE<0.05	Mean Cell-subtype Prop. (%)
Neutrophil	2151	0.13	0.23	0.41	620 (28.8)	65.0
CD4^+^T	2902	0.62	0.21	0.09	158 (5.4)	13.4
CD8^+^T	1871	0.14	0.14	0.48	479 (25.6)	6.1
Nat. Killer	3301	0.13	0.21	0.56	882 (26.7)	2.4
CD19^+^B	12973	0.78	0.2	0.03	635 (4.9)	3.0
Monocyte	2772	0.91	0.29	0.02	117 (4.2)	5.4
Eosinophil	7968	0.75	0.24	0.03	381 (4.8)	3.8
Lymphocyte-I	103035	0.15	0.15	0.46	25920 (25.2)	25.0
Myeloid-I	102934	0.12	0.15	0.56	28298 (27.5)	74.2
Lymphocyte-II	69455	0.14	0.16	0.52	17790 (25.6)	22
Myeloid-II	28891	0.18	0.2	0.44	6054 (21)	68.8
Pan-T	2911	0.31	0.17	0.21	353 (12.1)	19.6

Summary statistics are compiled here for each robust estimation panel. Observed is the LR estimate, expected value is the cell-sorted estimate. Mean cell-subtype proportions are calculated from FACS estimates. AE: Absolute error (CpG-specific), MAE: Mean Absolute Error (averaged over panel), MMCE: Mean Mixed-cell Error. *R*^*2*^: Coefficient of determination.

We compared the robust CpG panels to three CpG panels compiled for cell-type proportion estimation. These were the 300-CpG panel compiled using the IDOL algorithm [20], the 333-CpG panel present in the epiDISH package [21], and a 600-CpG panel identified using the algorithm in the estimateCellCounts function in the minfi package[22]. We found that 94.3% of IDOL CpGs were found on at least one panel, while 99.0% and 96.8% were found for the CpGs in the minfi panel and the epiDISH panel respectively. A full breakdown for each cell-subtype panel is presented in S2 Table.

### Biological relevance of the robust CpG panels

**Fig 2:**
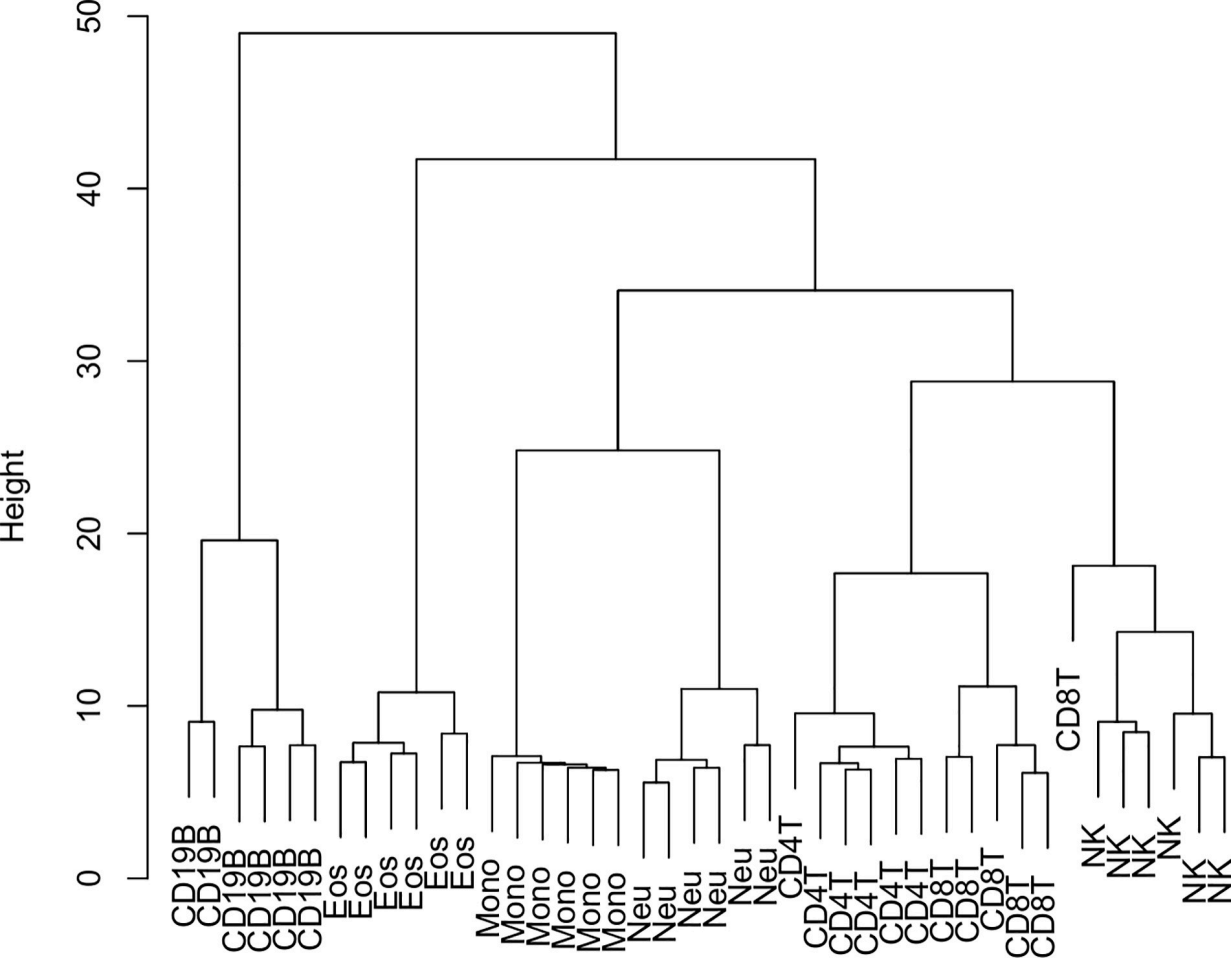
Hierarchical clustering of cell-sorted data from robust panels. Tree represents an unsupervised clustering of cell-sorted sample data from only the CpGs found in the robust panels for the base cell-subtypes. Terminal nodes correspond to single samples. Each sample is labelled by the type of cell-subtype to which it corresponds.

Hierarchical clustering of the cell-sorted samples based on the derived, robust CpG panels resulted in samples of the same cell-subtype clustering together (see Fig 2). There was a relatively weak resemblance to the hematopoietic lineage, although when we limited this analysis to the top 1000 CpGs from each robust CpG panel ranked by *t*-test p-value, the phylogeny aligned almost perfectly with expected lineages, with the exception of a single CD8^+^T sample (see S1 Fig). This CD8^+^T sample had more hemimethylated CpGs than the other CD8^+^T samples, which may indicate the presence of impurities in this sample (see S2 Fig).

A pathways enrichment analysis using the online toolkit WebGestalt GSAT showed a correspondence between identified CpGs and biological pathways for each cell type. For example, CpGs specific to CD4^+^T mapped to Th1,2,17 cell differentiation and T cell receptor signalling pathways (PFDR < 0.0005). These findings suggest that the CpG selection algorithm selected sites that were mostly of biological relevance and were plausible candidates for the estimation of cell-subtype methylation estimation in EWAS (see S4 and S5 Files for complete CpG and gene lists).

### Estimation performance of the cell-specific and lineage group CpG panels

The smallest Mean Absolute Error (MAE) was calculated in the Neutrophil, CD8^+^T, CD56^+^ NK, and Myeloid-I panels (Table 1, MAE = 0.10 for all), and the highest error was found in the CD19^+^B, Monocyte and Eosinophil panels (MAE = 0.6, 0.69, 0.59). The panels with the best panel performance were Natural Killer and Myeloid-I (*R*^*2*^ = 0.562; 0.563 respectively), while the worst performance was seen in the CD19^+^B, Monocyte, and Eosinophil panels (*R*^*2*^ = 0:034, 0:023, and 0:032 respectively). For CD4^+^T, CD19^+^B, Monocytes, Eosinophils, and Pan T, the larger MAE compared with MMCE indicated that linear regression failed to reduce the error from a zero-mean model. Lymphocyte-II showed no difference between the two values. In general, a large value of *R*^*2*^ between the LR estimate and cell-sorted estimates corresponded with an improvement of MAE over MMCE, while low *R*^*2*^ corresponded with a higher MAE than MMCE.

To more closely examine estimation error for sites known to be biologically linked, we examined 5 CpGs (cg00219921, cg04329870, cg08506127, cg19410791, cg25939861) known to be specific to the CD8A gene (see S3 Fig). These sites were selected for the large cell-sorted estimate in the cell-sorted data between the methylation of CD8^+^T and the other cell-subtypes, which meant they were a natural example to study a small number of cell-type specifically methylated CpGs. All were considered accurately estimated using the *D-*heuristic, but the absolute error for these CD8^+^ sites ranged from 0.01 (cg08506127) to 0.48 (cg19410791).

At the cell-subtype level, the total number of CpGs that yielded a calibrated estimate that was within 5% of the expected value was 3272 (9.6% of all cell-subtype associated sites). For all single cell-subtypes, only a small proportion of the reduced panel had estimates within 5% of the cell-sorted estimate (all < 30%). The proportion of accurately estimated CpGs ranged from low (5%) for CD4^+^T, Eosinophils, Monocytes and CD19^+^B to moderate for CD8^+^T, Natural Killers, and Neutrophils (25%).

There was no indication that average cell-fraction was related to the performance of cell-subtype specific methylation regression, since both CD8^+^T and Natural Killers are uncommon but had very good relative performance. The performances of large cell-subtype groupings were consistently high.

### Robust CpG panels are validated using second dataset

In order to determine whether the robust CpG panels were tied to particular dataset, we used a second dataset with both mixed-cell and cell-sorted samples (GSE Accession number: GSE88824). This dataset also contains female subjects, which provide a validation of the panels for data sets of both sexes. The same method as above was used to find robust CpG panels in this validation dataset and the contents compared by calculating the percentage of CpGs from the original panels, which were also found in the corresponding validation panel. This percentage ranged from 50% in Lymphocyte-II panels to 98% for the Neutrophil panel. The size of the robust panels varied, as did the percentage of accurately estimated CpGs in each panel. Notably, the CD8+T panel showed a large percentage (>25%) of accurately estimated CpGs in both the original and validation datasets compared to the 3% that would be expected under random chance. Complete corresponding figures and tables for the validation dataset are found in S1 Table, S4 Fig and S6 File.

## Discussion

Methylation levels at the individual CpG level are known to vary among whole blood cell-subtypes. Given that many EWASs utilise methylation data derived from mixed-cell samples (whole blood or PBMCs), a statistical estimation approach is the only option available for cell-specific analyses. The linear regression modelling approach has been used previously for estimation of cell-type specific methylation and gene expression in brain tissue but not in blood. This paper critically analysed the performance of linear regression estimation for estimating differences in cell-subtype methylation using mixed-cell methylation data. For the analysis, we examined a publicly available 450K methylation data set from 6 healthy males in which data was available from both mixed and cell sorted samples from the same individual [13]. Although this design was small in terms of sample size, it enabled us to directly compare the regression estimates derived from mixed-cell signal to the expected values derived from the cell sorted samples.

It should be noted that screening for SNPs in the cell panels identified that 27% of CpGs were collocated with SNPs. It is possible that the poor estimation performance at a substantial proportion of CpGs was the result of SNP effects. A measured approach for screening estimation panels might be to first screen SNPs by their minor allele frequencies to remove the most common, then treating the results of more rare SNPs with appropriate suspicion. Given the known interactions between single nucleotide variation and local CpG methylation, it may be necessary to adopt models with random effects to appropriately model genetic relatedness between samples. Further, it is likely that the estimation problem described could be addressed better by models which jointly estimate cell-type methylation differences at multiple CpG loci with the genomic context as a locus-specific predictor of methylation as well as accounting for relatedness.

The results for the entire epigenome indicate that estimates from linear regression can have magnitudes larger than 1, even though differences in cell-type means cannot. In this paper we adopted a *post hoc* calibration approach, however future methods could adopt a *post hoc* correction similar to how negative proportion estimates are corrected to 0 in Houseman’s proportion estimation method [19]. Another option may be to fit the linear regression subject to a set of constraints.

That larger cell-subtype groupings tended to have consistently good estimation performance indicates that cell-subtype methylation estimation should be performed in accordance with the information content of the data. For small samples sizes, the Lymphocyte-I and Myeloid-I groups may be distinguished, and adding more samples may allow for distinguishing between cell-subtypes within these groups. Additionally, while not possible for this analysis due to the small sample size, in a larger cohort extending the model from simple linear regression to more coefficients could be useful, particularly in CpGs where there are multiple methylation levels between cell-subtypes.

Since the number of samples in this study is small (n = 6 for each cell-subtype methylation estimate), there is an interest in determining how performance would improve as the sample size increases. As sample size increases one would expect the standard error of cell-subtype methylation estimates to decrease and thus correlate better with cell-sorted estimates in the group-wise case. In the site-wise case, the calibration phase would have less associated error and the absolute error would decrease. This benefit is twofold, as lower absolute error would mean a site is more likely to be accurately estimated, but the increased power of the *D* heuristic test would allow better discarding of sites with specific biases.

The samples acquired for analysis comprised healthy adult males, so there is likely to be some sensitivity of these panels to the age and sex distribution of a study’s subject group. This sensitivity was quantified in the case of sex, finding that a majority of CpGs in the original robust CpG panels were also seen in the validation panels, derived from a mixed sex subject pool. Therefore, there is some indication here that the original panels are robust and reliable for datasets of different sex distributions.

This analysis relies on scarce cell-sorted data to produce an independent and assumed accurate cell-sorted (expected) value for comparison with the regression estimate, so it is restricted to a very small number of samples. Given this restriction, simulation of methylation from heterogeneous tissue is likely the best way to explore the power of this validation; however, there are significant challenges in faithfully reproducing both the mixing process and cell-subtype methylation levels *in simulo*.

There is evidence that simply using the heterogeneous tissue without accounting for cell-subtype heterogeneity has the effect of increasing false positives, which may lead to spurious associations [23]. Consequently, a great amount of research has been published on the problem of correcting EWASs for cell-subtype heterogeneity (e.g [24]). While this type of analysis is related to this work, there is a fundamental difference in the type of inference being conducted. While the goal of Cell-type Specific Methylation Estimation (CSME) is to infer the methylation (and thus methylation differences) of specific cell-subtypes, the goal of heterogeneity correction is to infer methylation differences between two groups, corrected for potential differences in the cell-subtype compositions.

The method evaluated in this study, linear regression CSME, does not itself present an alternative to traditional EWAS, because it does not explicitly model any phenotypic relationship with methylation. The use of linear regression CSME extends naturally however to the context of multiple groups defined by different phenotype, exposure, or disease of interest. By estimating group-wise difference in beta-value methylation between the cell-type of interest and the other cell-types, one can then compare these estimates between the two groups where a discrepancy in the difference would imply a cell-type specific effect at the given locus, with an accompanying measure of significance based on standard errors. Given the precision of the group-wise difference estimate would affect the sensitivity and specificity of a test comparing between groups, the results of this study have implications for cell-type specific EWAS where linear regression is used.

These results have implications for the prospect of using linear regression estimation in EWAS to find CpG associations at the cell-subtype level. While these results were for a single group, meaning only a single group mean needed to be estimated, we can easily apply a case-control EWAS design requiring two group means to be estimated. An EWAS with a quantitative trait requires an interaction effect to be estimated; therefore a recommendation for using linear regression methylation estimation in this context is to ensure the cell-subtype of interest can be accurately estimated for a single control group before proceeding with an EWAS specific to that cell-subtype. Our results provide some indication of the cell-specific sites and degree of accuracy that can be expected when estimating group-level cell-subtype methylation for an EWAS design.

## Supporting information

S1 File Derivation of the linear regression deconvolution model from simple assumptions. (PDF)

S2 File Simulation demonstrating statistical calibration correcting for scale and location shifts. (PDF)

S3 File Detail for the use of *t*-distribution as the distribution of *D*. (DOCX)

S4 File Complete CpG lists for robust panels. (XLSX)

S5 File Complete Gene lists for robust panels. (XLSX)

S6 File Complete CpG and gene lists for robust panels generated from validation dataset. (XLSX)

S1 Fig Hierarchical clustering of cell-sorted data using top 1000 CpGs from each robust panel from base cell-subtypes. (PDF)

S2 Fig Densities of CD8+T beta-value methylations for top 1000 CpG panel. The red coloured line corresponds to the outlier CD8+T sample which did not cluster with the other CD8+T samples in the hierarchical clustering.
(PDF)

S3 Fig Box-plots of beta-values from cell-sorted data at 5 CpGs on the CD8A gene. Plots show the beta-values of CD8T sorted samples at these CpGs are much lower than the beta-values from samples sorted to other cell-types.
(PDF)

S4 Fig Corresponding plots for validation dataset. A) The LR estimate versus the cell-sorted estimate derived from the validation dataset for all CpGs, performed for each cell-subtype and lineage grouping present in the cell-sorted data. Note the Myeloid-alt grouping is equivalent to the Myeloid-I grouping minus the Eosinophil cell-subtype, which is not present in the validation data. B) Hierarchical clustering for cell-sorted data using only CpGs from robust panels of the base cell-subtypes, and C) clustering using only the top 1000 CpGs from each robust panels of base cell-subtypes.
(PDF)

S1 Table Estimation performance over the robust panels for cell-subtype and lineage groupings for the validation data set. AE: Absolute Error (for single CpG), MAE: Mean Absolute Error (over CpG panel), MMCE: Mean Mixed-cell Error.
(DOCX)

S2 Table Comparison of CSME robust CpG panels with the CpG panels derived by 3 cell-type proportion estimation methods: IDOL, minfi, and epiDISH. Percentages are calculated relative to the size of the cell-type proportion estimation panels.
(DOCX)
