# Supplementary material for: Critical evaluation of linear regression models for cell-subtype specific methylation signal from mixed blood cell DNA
Source: PLoS One. 2018 Dec 20;13(12):e0208915. doi: 10.1371/journal.pone.0208915 (PMC6301777; doi:10.1371/journal.pone.0208915)
Supplement: S1 File — (PDF) [file pone.0208915.s001.pdf]

S1 File

1 Derivation of linear regression model

The following is a derivation of the linear regression model used in this paper based on the linear mixing process assumption.

The methylation level of at a given CpG for subject i, can be thought of as a measurement, with some level of noise, of the probability that a randomly drawn strand of DNA in the sample is methylated. Assuming the noise has mean 0:

$$\mathrm{E}(y_i) = \Pr(\text{strand is methylated})$$

It is known that cell-types exhibit different methylation levels, so we may brake this down by the law of total probability into the K constituent cell-types:

$$\Pr(\text{strand is methylated}) = \sum_{k=1}^{K} \Pr(\text{strand is methylated}|\text{strand in cell-type } k)\Pr(\text{strand in cell-type } k)$$

We can immediately recognise $\Pr(\text{strand in cell-type } k)$ as the relative proportion of cell-type k in the sample, so we may substitute this in as p_{ik}. Second, we assume that cell-type methylation levels are stable over subjects, so $\Pr(\text{strand is methylated}|\text{strand in cell-type } k)$ is labelled as x_k. We therefore have the equation

$$\mathrm{E}(y_i) = \sum_{k=1}^{K} x_k p_{ik}$$

which we can call the linear mixing process assumption. We can then include a noise term $\epsilon_i \sim \mathrm{Normal}(0, s)$ with standard deviation s:

$$y_i = \sum_{k=1}^{K} x_k p_{ik} + \epsilon_i.$$

When studying whether a particular cell-type k is differently methylated from the rest we may aggregate the other cell-types, forming a simple linear regression problem.

$$y_i = p_{ik} x_k + \sum_{q \neq k} p_{iq} x_q + \epsilon_i \qquad (1)$$

If we let $x_{-k} = \sum_{q \neq k} \frac{\bar{p}_q}{1-\bar{p}_k} x_q$ where $\bar{p}_k = \mathrm{E}(p_{ik})$, then the equation 1 can be written as:

$$\begin{aligned} y_i &= p_{ik}x_k + \sum_{q \neq k} p_{iq} \left(x_q - x_{-k} + x_{-k}\right) + \epsilon_i \\ y_i &= p_{ik}x_k + \sum_{q \neq k} p_{iq}x_{-k} + \sum_{q \neq k} p_{iq} \left(x_q - x_{-k}\right) + \epsilon_i \\ y_i &= p_{ik}x_k + \left(1 - p_{ik}\right) x_{-k} + \underbrace{\sum_{q \neq k} p_{iq} \left(x_q - x_{-k}\right)} + \epsilon_i \end{aligned}$$

If we look at the expectation of the under-braced term, we see that it has a mean of 0:

$$\begin{aligned} \mathrm{E}\left(\sum_{q \neq k} p_{iq} \left(x_q - x_{-k}\right)\right) &= \mathrm{E}\left(\sum_{q \neq k} p_{iq}x_q - \sum_{q \neq k} p_{iq}x_{-k}\right) \\ &= \sum_{q \neq k} \mathrm{E}(p_{iq})x_q - x_{-k} \sum_{q \neq k} \mathrm{E}(p_{iq}) \\ &= \left(1 - \bar{p}_k\right) x_{-k} - x_{-k} \left(1 - \bar{p}_k\right) \\ &= 0 \end{aligned}$$

By assuming that this term has Normal error with standard deviation s_{-k}, we can combine this with the noise as a new term for all unmodelled variation $e_i \sim \text{Normal}\left(0, \sqrt{s^2 + s_{-k}^2}\right)$. Therefore, we arrive at the simple linear regression equation:

$$\begin{aligned} y_i &= p_{ik}x_k + \left(1 - p_{ik}\right) x_{-k} + e_i \\ y_i &= x_{-k} + p_{ik} \left(x_k - x_{-k}\right) + e_i \\ y_i &= \beta_{0k} + p_{ik}\beta_{1k} + e_i \end{aligned}$$
