# Supplementary material for: Critical evaluation of linear regression models for cell-subtype specific methylation signal from mixed blood cell DNA
Source: PLoS One. 2018 Dec 20;13(12):e0208915. doi: 10.1371/journal.pone.0208915 (PMC6301777; doi:10.1371/journal.pone.0208915)

# Demonstration - Need for Calibration

*Daniel Kennedy*

*8 November 2018*

## Introduction

The following code is a simulation of whole blood methylation from ficticious cell-type methylation profiles which are correlated, and simulated proportions. The purpose of the simulation is to show linear regression estimates of the cell-sorted difference can show a position shift and/or scaling, and that this is easily corrected by a post-hoc statistical calibration.

We load the library `gtools`, set the experiment parameters:

```
set.seed(353)

library(gtools)
#Set number of samples (I),
#set number of loci (J), and the noise
#standard deviation:
I <- 8
J <- 1000
sigma = 0.05
```

We then simulate some relative proportions for the ficticious cell-subtypes:

```
#Simulate I relative proportion/cell-subtype fractions:
props <- rdirichlet(I, 4 * c(0.6,0.2,0.2))
```

We simulate some cell-type methylation profiles, such that the first two are correlated:

```
#Simulate cell-type 1 between 0 and 1.
c1 <- runif(J,0,1)

#Simulate cell-type 2 as highly correlated with c1.
c2 <- c1 + runif(J,-0.1,0.1)

#Simulate cell-type 3 as uncorrelated with c1 and c2.
c3 <- runif(J,0,1)

#Bind cell-sorted values into a matrix:
cell_sorted_vals <- rbind(c1,c2,c3)
```

Finally, we simulate some whole blood methylation profiles for the `I` samples:

```
#Use linear mixing assumption to simulated whole blood methylation.
y <- props %*% cell_sorted_vals + matrix(rnorm(I*J,0,sigma),I,J)
```

We calculate the cell-sorted differences from the cell-type methylation profiles directly, and then estimate them using linear regression:

```
#Calculate cell-sorted differences:
cell_sorted_diffs <- rbind(
  cell_sorted_vals[1,] - apply(cell_sorted_vals[-1,],2,mean),
  cell_sorted_vals[2,] - apply(cell_sorted_vals[-2,],2,mean),
  cell_sorted_vals[3,] - apply(cell_sorted_vals[-3,],2,mean)
```

```
)

#Estimate cell-type differences by coefficients using linear regression:
est.coefs <- rbind(
  (solve(t(cbind(1,props[,1])) %*%  cbind(1,props[,1])) %*% t(cbind(1,props[,1])) %*% y)[2,],
  (solve(t(cbind(1,props[,2])) %*%  cbind(1,props[,2])) %*% t(cbind(1,props[,2])) %*% y)[2,],
  (solve(t(cbind(1,props[,3])) %*%  cbind(1,props[,3])) %*% t(cbind(1,props[,3])) %*% y)[2,]
)
```

The plots show that the values exhibit the incorrect scaling, which is extreme in the case of Cell-type 2:

```
#Plot estimates versus cell-sorted differences.
par(mfrow = c(2,2))
for(i in 1:3){
plot(x = cell_sorted_diffs[i,], y = est.coefs[i,],
     xlab = "Cell-sorted Difference",
     main = paste0("Cell-type ",eval(i)),
     ylab = "Estimated difference")
abline(lm(est.coefs[i,] ~ cell_sorted_diffs[i,]),col = "red",lwd  = 2)
#print(summary(lm(est.coefs[i,] ~ cell_sorted_diffs[i,])))
abline(0,1,col = "green")
}
```

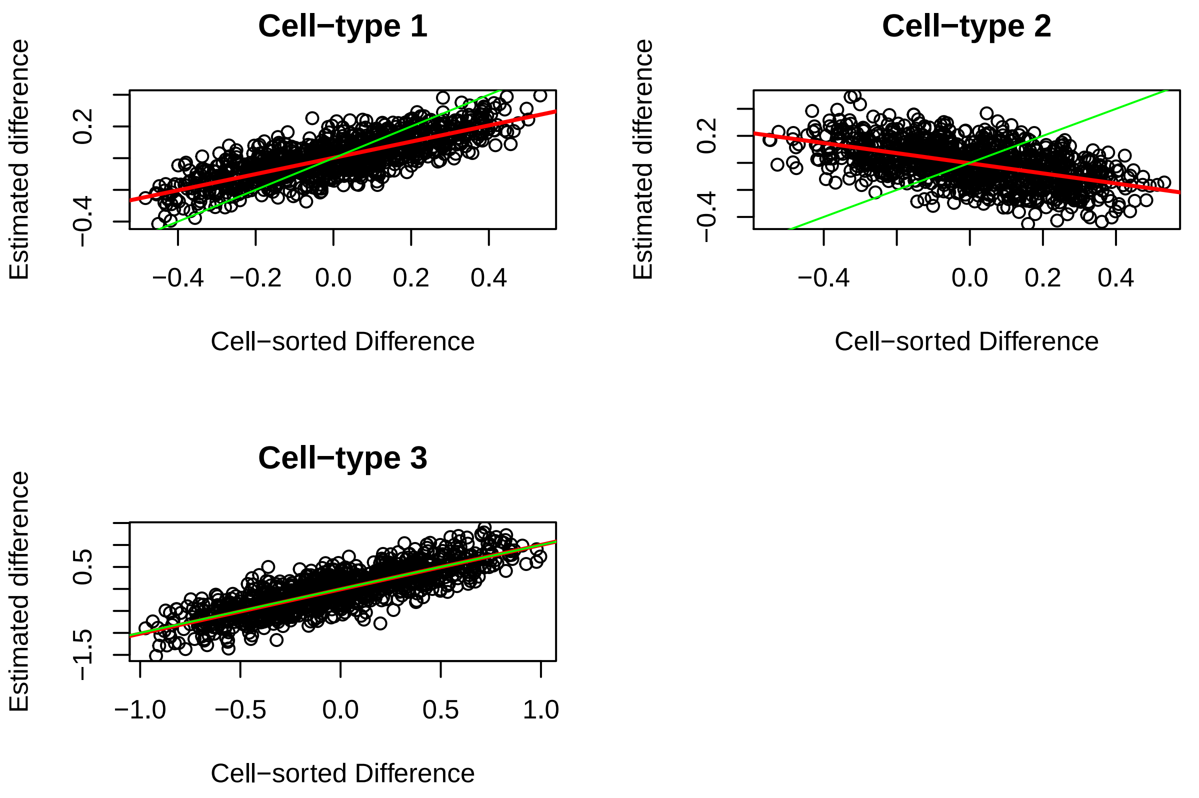


However, there is still a linear relationship between the estimate and the true value, which through a simple calibration, as in the main analysis, results in a good correspondence:

```r
#Create simple function to calibrate as in main analysis.
calibrate_y <- function(x,y){

  coefs <- coef(lm(y ~ x))
  return((y - coefs[1]) / coefs[2])
}

#Plot calibrated version.
plot(
  x = cell_sorted_diffs[2,],
  y = calibrate_y(x = cell_sorted_diffs[2,], y = est.coefs[2,]),
  xlab = "Cell-sorted Difference",
  ylab = "Calibrated estimated difference")
abline(
  lm(
    calibrate_y(
      x = cell_sorted_diffs[2,],
      y = est.coefs[2,]) ~
      cell_sorted_diffs[2,]),col = "red",lwd  = 2)
abline(0,1,col = "green")
```

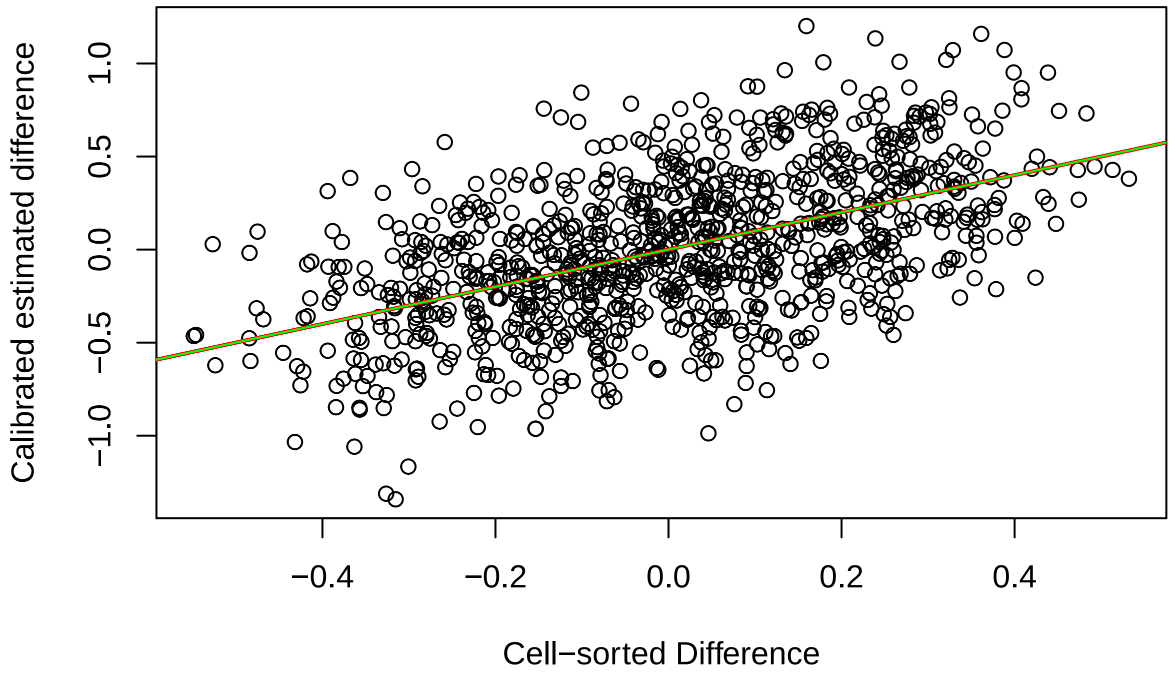

Supplement: S2 File — (PDF) [file pone.0208915.s002.pdf]
