# Supplementary material for: Critical evaluation of linear regression models for cell-subtype specific methylation signal from mixed blood cell DNA
Source: PLoS One. 2018 Dec 20;13(12):e0208915. doi: 10.1371/journal.pone.0208915 (PMC6301777; doi:10.1371/journal.pone.0208915)
Supplement: S3 File — (DOCX) [file pone.0208915.s003.docx]

**On the choice of distribution for *D***

Let *s* be the LR estimate, $c$ be the cell-sorted estimate, and *α* and *β* are the calibration parameters. Therefore the calibrated LR estimate is $\frac{s-\alpha}{\beta}$ and the standard error of the LR estimate is $\left| \frac{1}{\beta} \right|\mathrm{SE}(s)$. Therefore, the $D$-heuristic in terms of *s* is

$$D=\frac{\frac{s-\alpha}{\beta}-c}{\left| \frac{1}{\beta} \right|\mathrm{SE}(s)}=\frac{s-\alpha-\beta c}{sign(\beta)\mathrm{SE}(s)} ,$$

where *c* is the cell-sorted value. Therefore, transforming the cell-sorted estimate $s_{0}=\alpha+\beta c$,

$D=sign(\beta)\left( \frac{s-s_{0}}{\mathrm{SE}(s)} \right)$.

Given *s* is a slope parameter in a linear regression, the null distribution of $\frac{s-s_{0}}{\mathrm{SE}(s)}$ is a *t*-distribution with $n-2$ degrees of freedom. Given *D* is equal to this value up to the sign, as long as the test is two-sided, the *p*-value from a *t*-test is valid. Specifically,

$$p=\Pr\left( T>D \right)$$

with $T\sim t(n-2)$.
