# Supplementary figures and images for: Critical evaluation of linear regression models for cell-subtype specific methylation signal from mixed blood cell DNA
Source: PLoS One. 2018 Dec 20;13(12):e0208915. doi: 10.1371/journal.pone.0208915 (PMC6301777; doi:10.1371/journal.pone.0208915)

Height

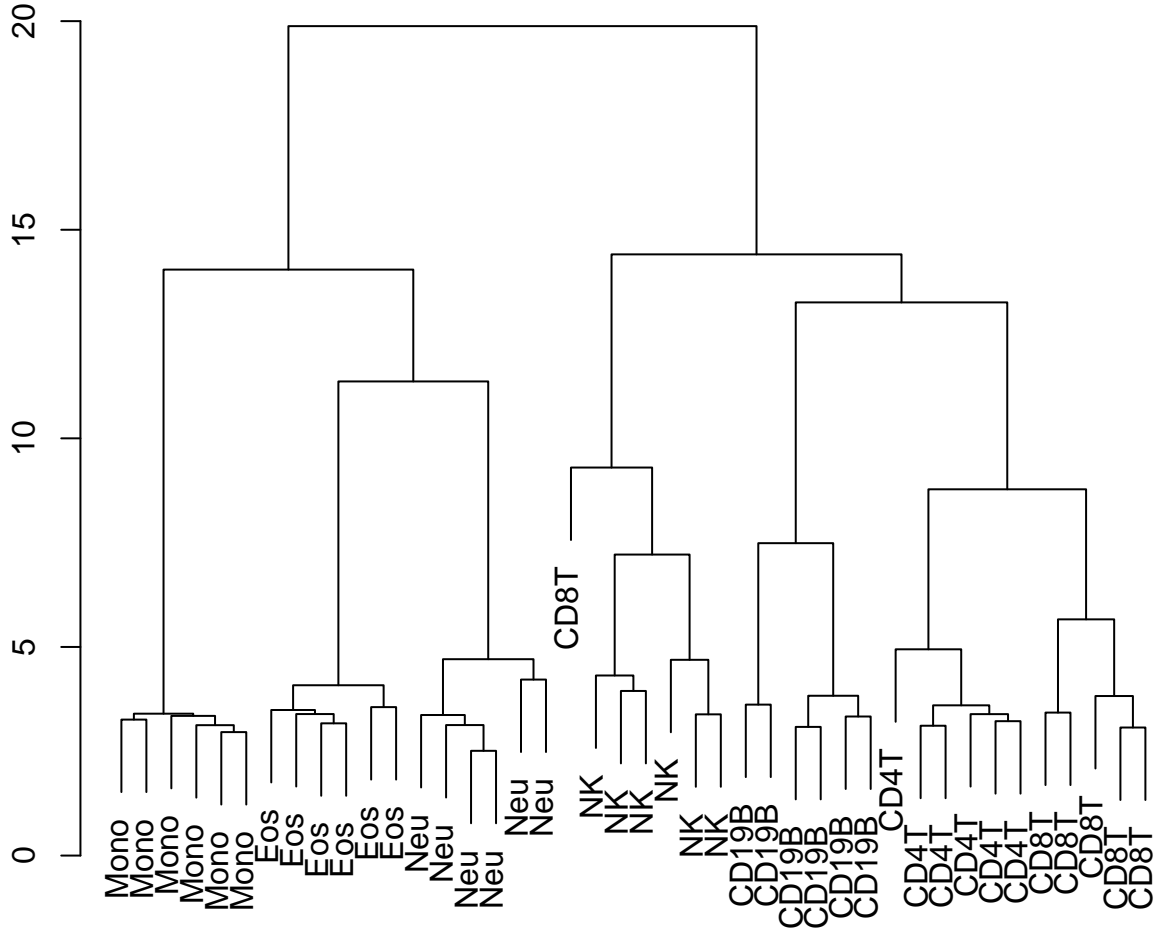

Supplement: S1 Fig — (PDF) [file pone.0208915.s007.pdf]

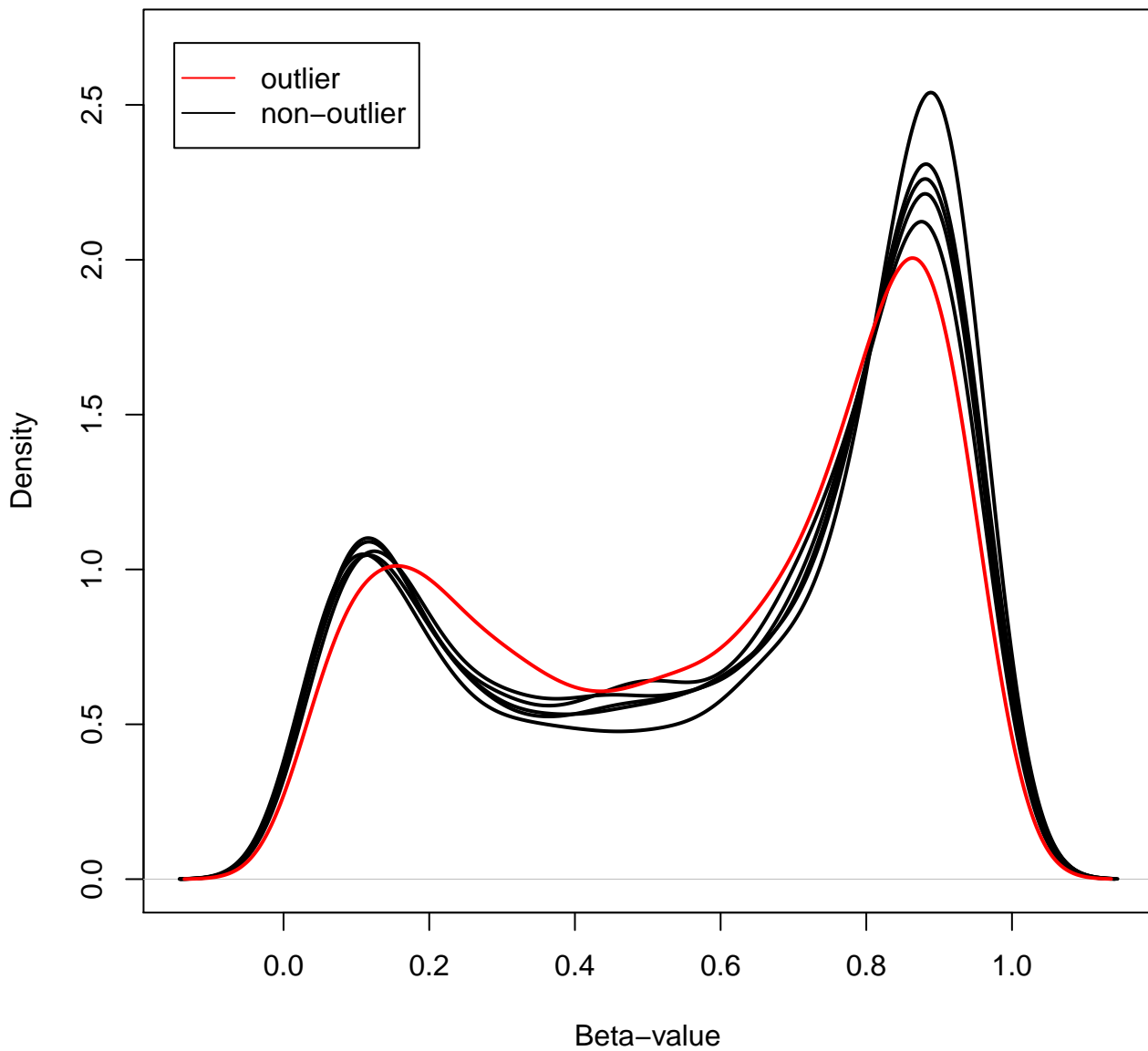

Supplement: S2 Fig — The red coloured line corresponds to the outlier CD8+T sample which did not cluster with the other CD8+T samples in the hierarchical clustering. (PDF) [file pone.0208915.s008.pdf]

Beta-value

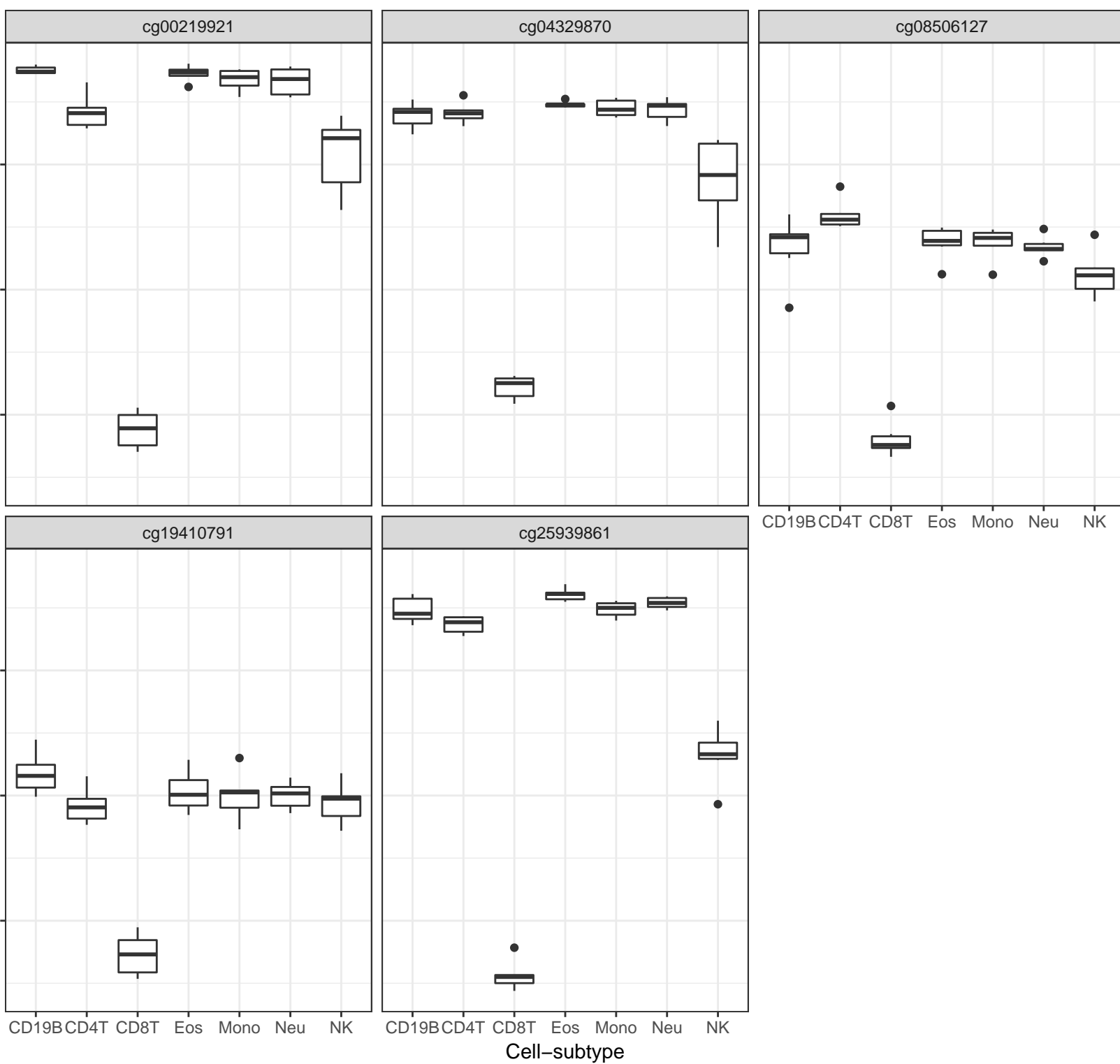

Supplement: S3 Fig — Plots show the beta-values of CD8T sorted samples at these CpGs are much lower than the beta-values from samples sorted to other cell-types. (PDF) [file pone.0208915.s009.pdf]

A

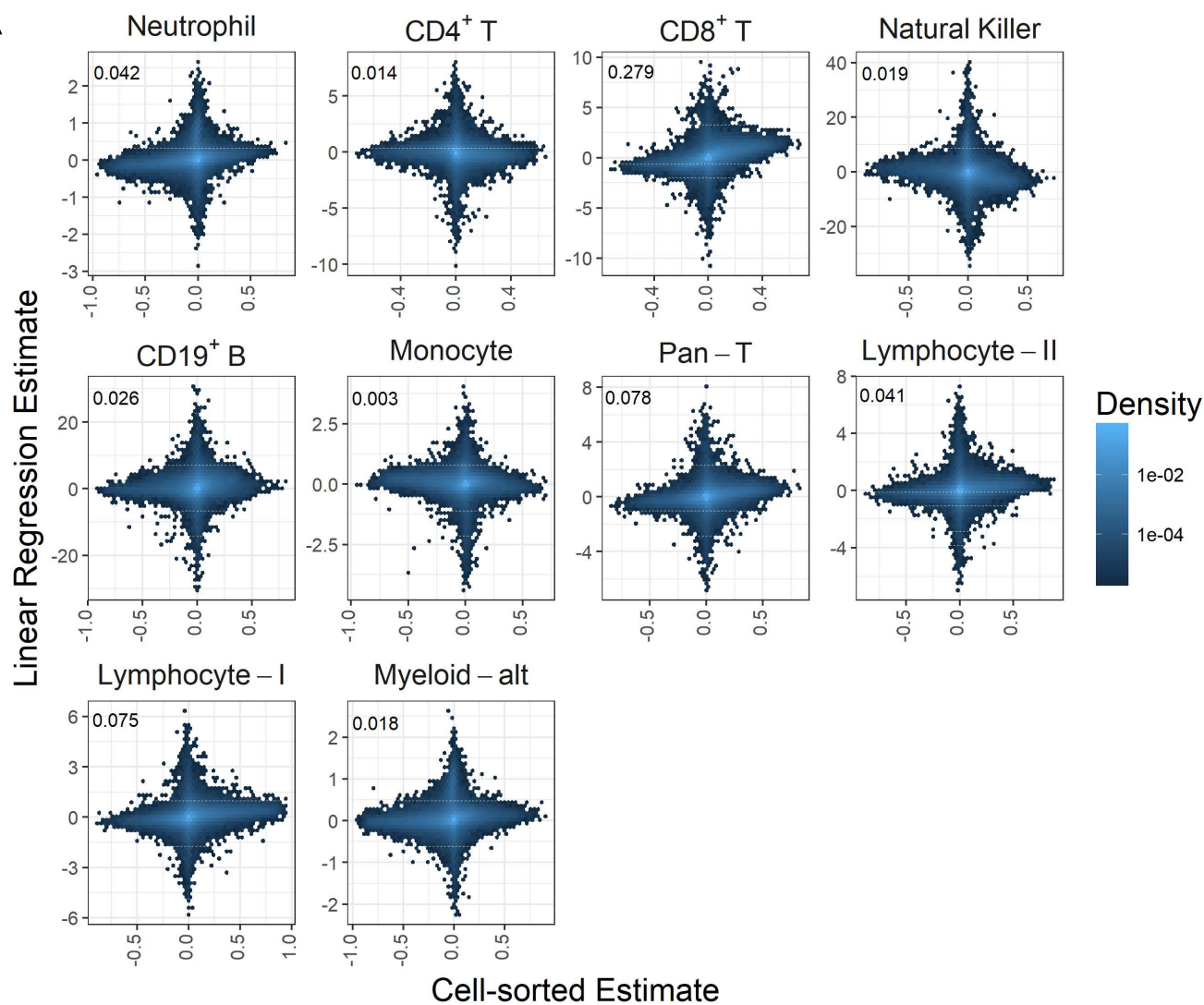

B

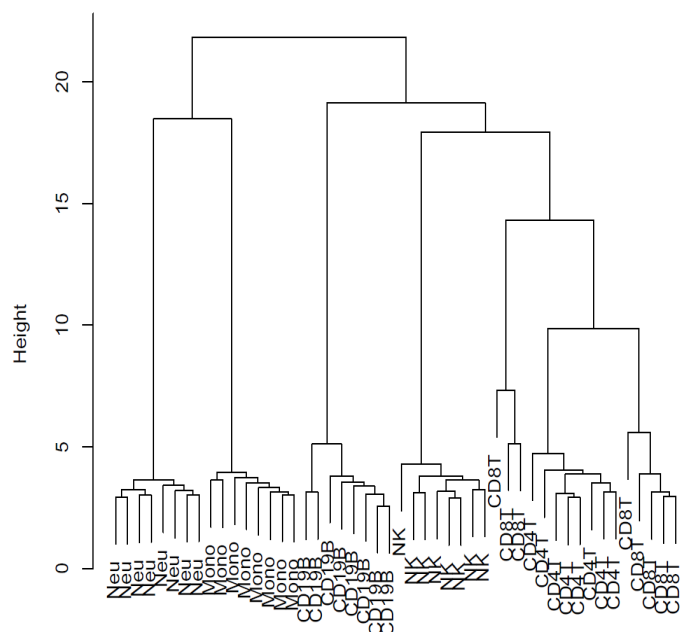

C

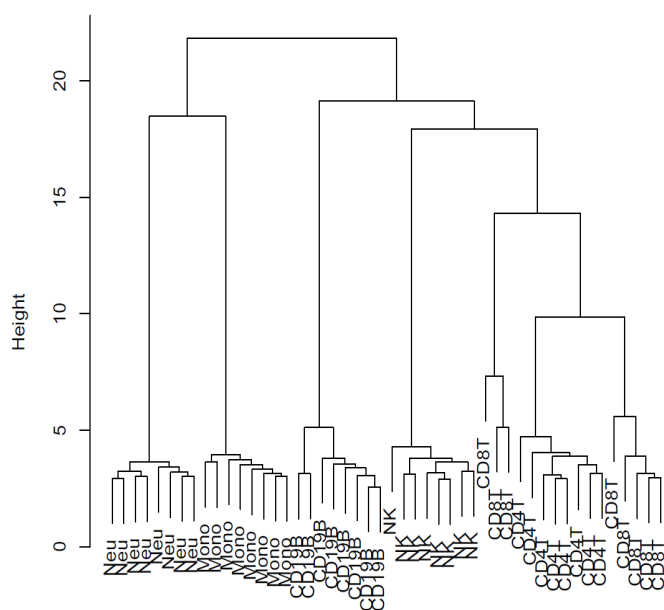

Supplement: S4 Fig — A) The LR estimate versus the cell-sorted estimate derived from the validation dataset for all CpGs, performed for each cell-subtype and lineage grouping present in the cell-sorted data. Note the Myeloid-alt grouping is equivalent to the Myeloid-I grouping minus the Eosinophil cell-subtype, which is not present in the validation data. B) Hierarchical clustering for cell-sorted data using only CpGs from robust panels of the base cell-subtypes, and C) clustering using only the top 1000 CpGs from each robust panels of base cell-subtypes. (PDF) [file pone.0208915.s010.pdf]
