# Supplementary material for: Critical evaluation of linear regression models for cell-subtype specific methylation signal from mixed blood cell DNA
Source: PLoS One. 2018 Dec 20;13(12):e0208915. doi: 10.1371/journal.pone.0208915 (PMC6301777; doi:10.1371/journal.pone.0208915)
Supplement: S1 Table — AE: Absolute Error (for single CpG), MAE: Mean Absolute Error (over CpG panel), MMCE: Mean Mixed-cell Error. (DOCX) [file pone.0208915.s011.docx]

| Panel | Panel Size | MAE | MMCE | Observed versus Expected (*R^2^*) | Number (%) with AE<0.05 | Mean Cell-subtype Prop. (%) |
| --- | --- | --- | --- | --- | --- | --- |
| Neutrophil | 15362 | 0.25 | 0.26 | 0.30 | 2259 (14.7) | 52.9 |
| CD4^+^T | 4623 | 3.3 | 0.21 | 0.00 | 54 (1.2) | 14.2 |
| CD8^+^T | 14177 | 0.08 | 0.16 | 0.76 | 6086 (42.9) | 8.7 |
| Nat. Killer | 5585 | 0.33 | 0.24 | 0.26 | 546 (9.8) | 2.8 |
| CD19+B | 17666 | 0.29 | 0.16 | 0.24 | 2060 (11.7) | 3.1 |
| Monocyte | 4086 | 0.74 | 0.31 | 0.06 | 188 (4.6) | 10.1 |
| Myeloid-alt | 60757 | 0.4 | 0.19 | 0.15 | 5013 (8.3) | 62.9 |
| Lymphocyte-I | 60700 | 0.24 | 0.19 | 0.31 | 9755 (16.1) | 28.9 |
| Lymphocyte-II | 39786 | 0.28 | 0.2 | 0.25 | 5418 (13.6) | 25.8 |
| Pan-T | 4631 | 0.24 | 0.19 | 0.31 | 690 (14.9) | 22.9 |

**S4 Table. Estimation performance over the robust panels for cell-subtype and lineage groupings** **for the validation data set**. AE: Absolute Error (for single CpG), MAE: Mean Absolute Error (over CpG panel), MMCE: Mean Mixed Cell Error.
