# Supplementary material for: Critical evaluation of linear regression models for cell-subtype specific methylation signal from mixed blood cell DNA
Source: PLoS One. 2018 Dec 20;13(12):e0208915. doi: 10.1371/journal.pone.0208915 (PMC6301777; doi:10.1371/journal.pone.0208915)
Supplement: S2 Table — Percentages are calculated relative to the size of the cell-type proportion estimation panels. (DOCX) [file pone.0208915.s012.docx]

|  | IDOL [20] |  | Minfi [21] |  | epiDISH [22] |  |
| --- | --- | --- | --- | --- | --- | --- |
|  | Number of CpGs in common | Perc. % | Number of CpGs in common | Perc. % | Number of CpGs in common | Perc. % |
| Neutrophil | 11 | 3.7 | 100 | 14.3 | 10 | 5.3 |
| CD4^+^T | 48 | 16.0 | 99 | 14.1 | 13 | 6.9 |
| CD8^+^T | 29 | 9.7 | 98 | 14.0 | 6 | 3.2 |
| Nat. Killer | 37 | 12.3 | 94 | 13.4 | 29 | 15.4 |
| CD19+B | 59 | 19.7 | 95 | 13.6 | 36 | 19.1 |
| Monocyte | 35 | 11.7 | 100 | 14.3 | 25 | 13.3 |
| Eosinophil | 21 | 7.0 | 91 | 13 | 17 | 9.0 |
| Pan-T | 49 | 16.3 | 99 | 14.1 | 13 | 6.9 |
| Lymphocyte-II | 111 | 37.0 | 280 | 40.0 | 106 | 56.4 |
| Lymphocyte-I | 123 | 41.0 | 316 | 45.1 | 102 | 54.3 |
| Myeloid-I | 122 | 40.7 | 314 | 44.9 | 102 | 54.3 |
| Myeloid-II | 61 | 20.3 | 191 | 27.3 | 40 | 21.3 |
| All Panels | 283 | 94.3 | 693 | 99.0 | 182 | 96.8 |

**S12 Table: Comparison of CSME robust CpG panels with the CpG panels derived by 3 cell-type proportion estimation methods: IDOL, minfi, and epiDISH.** Percentages are calculated relative to the size of the cell-type proportion estimation panels.
